# The development of the performance sex gap in track-and-field events across the lifespan

**DOI:** 10.3389/fspor.2025.1659762

**Published:** 2025-10-23

**Authors:** Barbora Balcarova, Arve Vorland Pedersen

**Affiliations:** Department of Neuromedicine and Movement Science, NTNU, Trondheim, Norway

**Keywords:** performance sex gap, development, track and field, athletics, women sports, top performance, scaling, sex difference

## Abstract

Due to sexual dimorphism, men outperform women in athletics, and thus they compete separately. Although the difference, called “the performance sex gap”, was thought to be stable across the lifespan, our research suggests that the gap changes with age. In our study, using publicly accessible databases, we collected data about top performances by men and women 5 to 100 years old in 18 track-and-field events sorted into three categories: running, jumping, and throwing. Our results suggest that the magnitude of the performance sex gap changes across the lifespan; it emerges in prepubertal children, widens among adolescents, stabilizes among senior athletes (i.e., 20–34 years old), and further increases among masters athletes (i.e., 35+ years old). Among seniors, the gap has been narrower in running events (i.e., approx. 10%) than in jumping events (i.e., approx. 15%). In throwing events, the gap differs considerably, largely due to the variable scaling of throwing implements based on sex and age group. Among masters, for instance, those implements are generally scaled down more for men than for women, which makes sex-based comparisons difficult. Beyond that, scaling in general is vaguely defined and varies across countries and events for children athletes (i.e., 5–15 years old). Altogether, our results indicate that not only physiology but also event-specific tasks and environmental constraints influence the performance sex gap. To ensure fair scaling, throwing implements used among masters women athletes may need to be downscaled to allow an appropriate comparison of how performance develops in both sexes.

## Introduction

In athletics, women and men compete separately due to biological sex-based differences stemming from sexual dimorphism (1, 2). The total difference, dubbed the “performance gap” (3), “sports sex gap” (4), or simply “sex difference” (5–9), previously termed also as “gender gap” (10), can be represented as the difference between men's and women's performance measured in percentages (1, 10–13). In this article, we used the term “performance sex gap”, which highlights the athletic performance and refers to the sexual dimorphism. Except in recent years, the performance sex gap has narrowed over time (4, 10, 13), especially as women's athletics and training facilities have improved (14). Over the years, more events for women have also been added to the Olympic Games, which has helped to promote women's athletics and improve their conditions for training. Beyond that, the number of women participating in the major events is gradually increasing toward becoming equal with men; at the Winter Olympics in Tokyo in 2022, 44.7% of competitors were women, and at the Summer Olympics in Paris in 2024, half of the competitors were women for the first time ever (15). Although the rapid narrowing of the performance sex gap has even prompted speculation that women might outperform men in the future (16), such speculation was based on rather shaky ground, based on extrapolations of historic data showing the rapid improvement in the world record for the women's 100 m sprint. In fact, several studies have predicted that the gap will widen slightly in men's favor due to subtle improvements in men's performance (4, 17). Overall, however, the performance sex gap seems to have stabilized and even plateaued (10, 11, 18).

### Performance sex gap

The abundance of evidence about the performance sex gap's magnitude among elite athletes and event-based variations shows that men dominate in most strength-, power-, and speed-based events (10, 11, 18, 20). However, most such studies have investigated adult athletes (i.e., senior athletes, defined as aged 20 years and older by World Athletics) in their main career (4, 10, 19, 20), knowledge about younger athletes' performance remains limited (5–7, 21–24), as does knowledge about older athletes (i.e., 35–100 years old), dubbed “masters athletes” (25–28). To date, research has generally suggested that the performance sex gap varies between different periods in life due to age and the different development of the sexes. Even so, no study has compared the performance sex gap's development across the lifespan.

### Age

Individual performance changes across the lifespan (29), with a steep increase during childhood and adolescence, a peak during young adulthood, and a decrease in older age (1, 25). Although small, differences in performance occur even among child athletes in running, jumping, and swimming events (30). Research has revealed a performance sex gap of around 5% before the age of 12 years (5, 7, 21). Plus, in more recent studies, prepubertal boys outperformed girls in two age categories, 8 and under, and 9–10 years in running events (i.e., 100 m, 200 m, 400 m, 800 m and 1,500 m) with around 5% (22), while the gap in swimming events was smaller (i.e., between 1% and 3%) (30), as also reported elsewhere (23). Interestingly, however, even though the age of competitors was not specified, in 8 of 12 events in swimming, the top performance was set by a girl (23), similarly found by Senefeld et al. (31) in whose study the top five girls outperformed boys by 3% among children 10 years old or younger. By contrast, when a larger sample was considered, no performance sex gap emerged (31). Apparently, the top-performing girls in an age cohort under ten years old can outperform any boy; however, on a larger scale, the boys are faster, or else the difference is not significant (23, 31). Compared with elite child athletes, the gap among children in the general population is slightly greater. In a study of 3,621 prepubertal children, boys were significantly faster than girls—by 7.3%–10.7%—on a 1,600 m run (24). One of the explanations of boys' better performance may be connected to greater muscle mass and muscle strength (32, 33), along with other factors reported in Joyner et al.'s (30) recent review, including girls' lower engagement in physical activity or increased growth velocity associated with hormonal changes during infancy and boys' higher engagement in jumping and running while playing (30). However, because the research on athletic performance and somatic composition among children has yielded inconsistent results, more studies are needed (34).

Beginning at 12 or 13 years of age—that is, when boys enter puberty—the performance sex gap increases (5, 7, 21) to approximately 10% in running events and to nearly 20% in jumping events, reaching the adult performance sex gap levels before the age of 18 (5). Such changes correspond well with physiological and anthropometric variables such as height, weight, and body mass index; being alike in children less than 13 years old, but changing at the age of 14 or 15 years, when boys become taller and heavier, and girls develop thicker skinfolds (6). Thus, the magnitude of the performance sex gap increases by the age of 18, for running events (i.e., 10%–15%), and slightly more for jumping events (i.e., 18%–23%) (7, 21).

Among senior athletes (i.e., 20–34 years old), men outperform women in various events (4, 10, 19), but each sex achieves peak performance (e.g., best race time or performance score) at different ages. For example, women peak earlier than men in explosive sporting events (20) and throwing events (35) and as much as two years earlier in swimming events (29). However, performance declines with age as senior athletes become retired athletes, also called masters athletes, a term defined by the competition rules of World Athletics as “any athlete who has reached their 35th birthday” and competes in any of five-year age groups from 35 to 100 years of age. In the early masters period—that is, in an athlete's mid- to late 30 s—performance remains similar to that of senior athletes (25) while further decline is based on multifactorial changes connected with ageing. Such changes include, changes in the cardiovascular and pulmonary systems (36, 37), hormonal changes (36), changes in neuromuscular transfer velocities (38), and changes in body composition, including decreased muscle mass (38–40) and progressive increase of adipose tissue, especially in women (41).

The performance while ageing, changes according to the type of events. In brief, high-power activities such as weightlifting, the decline in masters athletes was pronounced more (25, 38), about 10% more in than in strength based activities in athletes 20–80 years old (38), in a cross-sectional study of 54 elite weightlifters the decline in power was 1,3% per year, while around 0,6% per year in isometric exercise (42). In endurance events such as cycling, rowing, swimming, and running, performance is relatively stable, even into old age (25). In fact, research has shown that masters athletes' performance has even improved in the past three decades (12, 13, 27, 28), partly due to the age group's increased participation in various master-level events (27, 28).

### Events

Among senior athletes, the performance sex gap clearly varies across events (9, 10, 17, 19). For instance, in running events, the gap is stable at approximately 10% and shows only slight variation from event to event (3, 10, 18, 19). In short-distance swimming events, the gap is similar, at approximately 12% (3, 17, 43). However, in endurance-dependent long-distance swimming events, the gap narrows, with women performing only 5%–6% poorer than men (10, 19), and in ultra-distance swimming races, women even outperform men (44, 45). The difference may be due to the higher amount of adipose tissue in women's bodies that helps with temperature resistance, increases buoyancy and improves swimming efficiency (45). Furthermore, the sexes differ in their metabolism of the working muscle (43) and muscle fatiguability, and while performing endurance exercise, women's metabolism relies more on lipid mechanisms than men's (46). Moreover, during the same exercise, women oxidize more fat in the muscle than men, while men rely more on carbohydrate oxidation and oxidize more protein than women (1, 47). However, the described mechanism is effective primarily in low-to moderate-intensity activities (approx. 40%–60% VO_2_ max) while, at high intensities, the carbohydrate oxidation dominates (48). Some authors have argued that the explanation lies in the different proportion of muscle type fibers, where type I fibers, more frequent in women muscles than in men's, contain more intramyocellular triacylglycerols. However, apparently, in women, both fiber types contain higher amounts of intramyocellular triacylglycerols than in men (47). Our study, however, did not have the requisite scope to cover all physiological processes exhaustively.

Across other events, the performance gap is larger, including in long-distance canoeing (i.e., >20%), and kayaking (i.e., 15%–17%), which require significant upper-body power (19). Also, a big gap has been found in the long jump, with women's performance being 18.8% poorer than men's (43).

### Sex

The performance sex gap is attributed primarily to physiological changes connected to sexual dimorphism (1, 2, 43), particularly due to the effect of testosterone, while the effects of female sex hormones and the role of the menstrual cycle are less impactful (2, 43). Prior to puberty, there is evidence of boys being stronger than girls (32, 33), even though the circulating testosterone before puberty is equal in both sexes, followed by pubertal changes in levels of testosterone that induce massive changes in boys' bodies (49). For girls, puberty starts earlier than for boys and features increased fat tissue and relatively small gains in muscle mass compared to boys (7), both of which cause girls' performance-related development to be more modest. As a result, their annual performance-related development is smaller compared to boys aged between 12 and 18 years (7). For boys, levels of circulating testosterone increase drastically in puberty— by around 30 times, in fact—and their bodies change into men's (1); their limbs become longer, and they develop more muscle mass, which results in more strength and power (43). Testosterone also affects the cardiovascular system; increased testosterone levels potentiate the production of hemoglobin, which is 12% greater in men than in women, and increase oxygen transport capacity (49), which, together with a proportionally larger heart and larger cardiac output in men (34, 50), are especially effective in endurance events (49).

With a higher percentage of muscle mass and longer limbs than women, men are physiologically stronger and develop greater explosive power (1, 18, 19). Power generation depends on two factors mainly—contraction velocity, faster typically in myosin type II fibers, and strength based on the size of the cross-sectional area—and in both cases men are at an advantage owing to a larger cross-sectional area of muscle and more myosin type II fibers (1, 47, 51). However, in certain environments, including water, women's physiological disadvantage (e.g., higher percentage of fat and relatively low muscle mass) is reduced due to differences in metabolism that women use, especially in ultra-endurance events (19). Although the difference narrows the performance sex gap (44), additional evidence on that count is needed.

Surprisingly, considering the increasingly equal participation rate of men and women in the major events (i.e., Olympic Games in Paris, 2024), the participation of men in studies on athletic performance heavily outweighs that of women, even though, the situation seems to have improved more recently (52). Research involving women participants only, however, remains rare and faces certain challenges (53). Women are underrepresented, specifically due to their menstrual cycles and hormonal changes that may affect women's performance, though the extent of the influence of those factors remains unknown (1, 47, 53). Moreover, while aging, women experience another hormonal swing due to menopause, the sudden end of the menstrual cycle that affects bone metabolism and protein synthesis into muscle mass, which reduces the amount of contractile tissue and increases the amount of intramuscular adipose tissue (39). Research has shown that postmenopausal women have poorer strength and balance than women of reproductive age (54, 55). Their response and adaptation to exercise is also altered (56). Thus, ageing causes the amount of muscle mass to drop in both sexes; however, the loss is faster in women after the fifth decade of life (39). In men, muscle mass is reduced progressively, by approximately 1% per year beginning in their late 30 s and can vary from person to person; however, despite low levels of testosterone in older age, many men stay asymptomatic, unaffected in their daily life (57, 58) and can maintain good performance into old age (59).

Sex-based physiological differences affect performance more in strength- and power-based events. Although the performance sex gap is narrower in endurance events, especially in running and lower-body endurance events, men still outperform women.

### Summary

Research on the performance sex gap has examined several athletic events, most often running and swimming events. However, most research has focused on a specific age period—senior athletes (8, 10, 19, 60)— and only a few studies have included youth athletes (5, 7, 21) or masters athletes (12, 25, 27, 61). When different age groups have been included in the same study (8), only a single event or group of events (e.g., running) has been investigated. Thus, the ways in which the performance sex gap develops across the lifespan remain unclear, particularly how much it increases and decreases with age and at what ages such changes occur. By extension, it is also unclear at what ages the gap is widest and when it is narrowest. Beyond that, due to large variations in the scaling of event-specific equipment, most notably the thrown implements in throwing events, it remains unknown whether comparisons of performance between the sexes are reliable for youth athletes and masters athletes, or even for senior athletes, and thus whether they provide a fair representation of performance-related development across the lifespan.

In response, in our study we aimed to describe how the performance sex gap changes in different age groups across the lifespan, notably in track-and-field events, grouped into the more general categories of running, jumping, and throwing. Because those event categories collectively represent a great deal of the variability in athletic behavior, our results would also provide more general knowledge about the development of the performance gap across the lifespan and illuminate related differences between the sexes.

## Materials and methods

### Data collection

From July 15 to December 20, 2022, data were collected from six publicly accessible athletics databases: European Athletics, International Age Records, Tillastopaja, Wellington Masters Athletics, World Athletics, and World Master Athletics (see Supplementary Appendix S1). No personally identifying or sensitive information was gathered during data collection.

Our dataset included all individual track-and-field events except hurdle events and steeplechase events, none of which had data for younger cohorts. In total, 18 athletic events were included and sorted into three categories: 10 running events (i.e., 100 m, 200 m, 400 m, 800 m, 1,500 m, 1 mile, 3,000 m, 5,000 m, 10,000 m, and marathon), four jumping events (i.e., long jump, triple jump, high jump, and pole vault), and four throwing events (i.e., shot put, hammer throw, javelin throw, and discus).

In the case of running events, the time spent running a specific distance was used as the main outcome. The value was displayed in hours, minutes, seconds, and hundredths of seconds, depending on the event's distance. For sprinting events (i.e., 100 m, 200 m, and 400 m) and middle-distance events (i.e., 800 m, 1,500 m, and 1 mile), the time in minutes, seconds, and hundredths was reported as m:ss.hh, whereas in long-distance running events (i.e., 3,000 m, 5,000 m, and 10,000 m), time in minutes and seconds was reported as mm:ss. Performance in the longest distance event, the marathon, was recorded in hours, minutes, and seconds as h:mm:ss. In the jumping and throwing events, the distance thrown (i.e., for the discus, hammer, javelin throw, and shot put) or length jumped (i.e., for the long jump and triple jump) or height jumped (i.e., for the high jump and pole vault) displayed in meters was used as the primary outcome.

The collected data included performance achieved (i.e., distance thrown, distance jumped, or completion time) and the athlete's sex and date of birth. The date and location of the event were also collected but not used for our immediate study's purpose. The top performance by an athlete in each cohort in males and in females was plotted and, altogether, the cohort-based performances revealed the development in performance across the cohorts. For athletes 5–34 years old, results for single-year age cohorts were available, whereas for athletes 35 years old and older, the cohort consisted of groups in five-year intervals (e.g., 35–39, 40–44, 45–49,…95–100); thus, the best result of any athlete in the cohort represented the cohort. The overall result was a chart showing the trend of the performance sex gap from children to masters athletes that compared both sexes. Only performance results obtained under formally accepted weather conditions (e.g., the wind did not exceed 2.0 m/s) were considered. Last, several performance results, particularly for children, were achieved at higher altitudes and are marked as such in the dataset.

Data processing consisted of initial preprocessing followed by conversion into MS Excel, with the men's data and women's data imported separately and sorted into age-based cohorts according to top performance. To enable a comparison between different categories of events (e.g., running × jumping × throwing), performance in time had to be converted to a simple unit. We thus used the fraction of the day, which is more accurate than simple rounding to a higher number and includes the fractions of seconds that matter in short-distance events (e.g., 100 m, 200 m, 400 m, and 800 m). Thus, for all running events, the recorded race times were converted into a fractional representation of the day, which facilitated their display in the chart. For jumping and throwing events, because distance in meters was the outcome, no data conversion was necessary, and the top performance in each cohort was used to construct the corresponding charts.

In throwing events, different weights of the throwing implements are used in competition over the lifespan, in youth and in masters athletes the weight has been reduced, while the main weight is used in senior athletes. In senior female athletes the weight of the throwing implement has been scaled down (Table 1) and in masters athletes, cohorts older than 50 years old use lighter weights to compensate ageing (Table 1). Among the younger age groups, because the rules specifying the throwing implements used by child athletes (i.e., 5–15 years old) lack uniformity and standardization, children of the same age worldwide throw implements of different weights and in different age categories. Some of those variations are summarized in Table 3.

**Table 1:** Weight of throwing implements used by adult athletes. The senior weight is used by athletes 20–49 years old, after which the master's implement is scaled. % MAX describes the percentage of senior's equipment (i.e., weight for masters women/weight for senior women and weight for masters men/weight for senior men). W/M% indicates the weight ratio between the sexes (i.e., women/men). For a detailed overview of scaling, see Supplementary Appendix S2.

Age	Shot put (kg)					Hammer (kg)					Javelin (g)					Discus (kg)				
	Men	% MAX	Women	% MAX	W/M %	Men	% MAX	Women	% MAX	W/M %	Men	% MAX	Women	% MAX	W/M %	Men	% MAX	Women	% MAX	W/M %
Main weight	7.26	100	4	100	55	7.26	100	4	100	55	800	100	600	100	75	2	100	1	100	50
50–59	6	83	3	75	50	6	83	3	75	50	700	88	500	83	71	1.5	75	1	100	67
60–69	5	69	3	75	60	5	69	3	75	60	600	75	500	83	83	1	50	1	100	100
70–74	4	55	3	75	75	4	55	3	75	75	500	63	500	83	100	1	50	1	100	100
75–79	4	55	2	50	50	4	55	2	50	50	500	63	400	67	80	1	50	0.75	75	75
80+	3	41	2	50	67	3	41	2	50	67	400	50	400	67	100	1	50	0.75	75	75

To examine the javelin throw in our study, only performances with the new javelin model were included to be consistent with rules established by the International Association of Athletic Federations in 1986. Beyond scaled weight, other features of event-specific equipment differ for women and men. In the javelin throw, for instance, the length of women's javelins is approximately 84% that of men's, and the center of gravity is shifted frontward by approximately 10 cm (i.e., equivalent to approx. 10%). Meanwhile, the diameter of the discus used by women is approximately 82% of that used by men; women's shot puts are approximately 86% the diameter of men's; and women's hammers are 98% of the length of men's and 84% of their diameter (see Supplementary Appendix S2).

### Data sample

The final sample of data included the top-performing athlete of each sex, the event, and the top performance, whether time or distance. Among children (i.e., from the age of 5) and adolescents and throughout the careers of senior athletes, the best result achieved by any athlete from each single-year age-based cohort was included. For masters athletes, who competed in five-year age-based cohorts, the top result for each cohort represented the cohort (e.g., 35–39, 40–44, but 95–100). The final dataset for each event included between 90 and 100 performances. In several athletes' records, the date of birth was missing in the original database; therefore, additional databases were consulted, primarily the Finnish database Tillastopaja, to gather that information. If still unavailable, then an extensive search was conducted using other databases and Google Search. When no source yielded the required data, the athlete's performance was excluded from the sample. Several other records in the database provided the year of birth without the specific date, and in such cases, efforts were made to pinpoint the date. However, in the few cases in which such attempts were unsuccessful, then the date of January 1 was used.

### Procedure

Our approach was explorative due to our study's purpose to describe performance-related development across the lifespan for both sexes and thus the corresponding development of the performance sex gap between men and women athletes. Our study design had elements of a natural experiment and of research involving big data. We used MS Excel for data preprocessing and transformative operations, as well as for simple analysis and for computing the performance sex gap using the following simple formula, which calculates the performance gap in a percentage:

$$\frac{PB_{M}-PB_{W}}{PB_{M}}\times 100$$

in which *PB_M_* represents the top performance among men and *PB_W_* the top performance among women. The formula was used to compute the performance gap in non-chronological events (i.e., jumping and throwing events). For chronological events (i.e., running events), in which the shortest time was the best performance, the gap was calculated using the following formula:

$$\frac{PB_{W}-PB_{M}}{PB_{W}}\times 100$$

Subsequently, data about the performance sex gap from all 18 events for age-based cohorts of athletes 5–100 years old were imported into Python Spyder, and a complex chart with polynomial regression was plotted. The outcome was a trendline of each event category (i.e., running, jumping, throwing) showing the performance gap development over the lifespan. Data variables included age, sex and events.

## Results

### Performance sex gap

**Figure 1:**
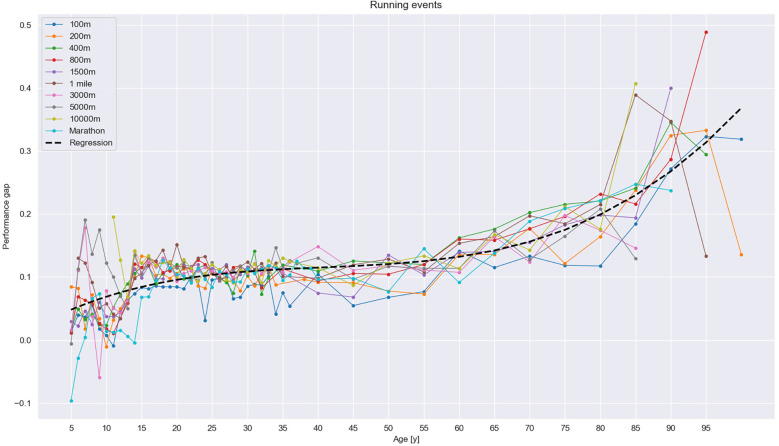
Performance sex gap in running events. The polynomial regression, with the equation 1.09 × 10^−6^x^3^ − 1.33 × 10^−4^x^2^ + 5.93 × 10^−3^x + 2.16 × 10^−2^, shows an increase in the performance sex gap among masters athletes.

**Figure 2:**
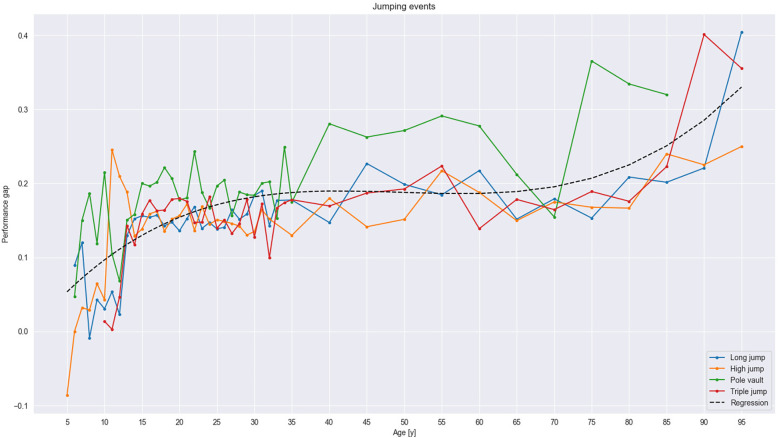
Trends in the jumping events. The polynomial regression curve, following the equation of 1.69 × 10^−6^x^3^ − 2.51 × 10^−4^x^2^ + 1.21 × 10^−2^x − 1.05 × 10^−3^, displays the same trend as in running events.

**Figure 3:**
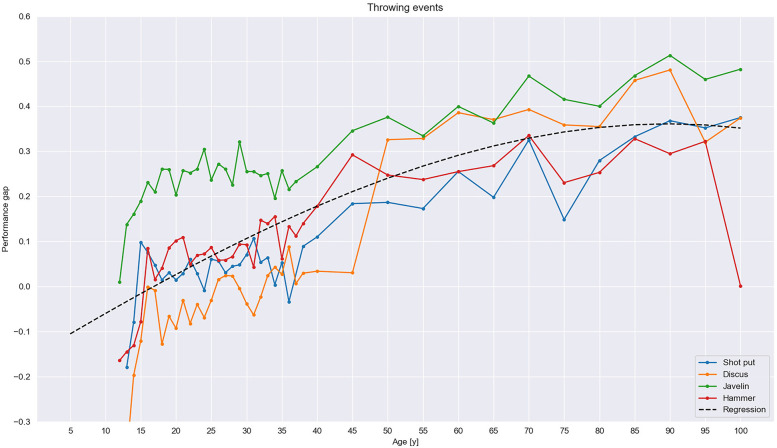
Throwing events. The performance sex gap in throwing events, following the polynomial regression line of −2.55 × 10^−7^x^3^ − 1.79 × 10^−5^x^2^ + 9.37 × 10^−3^x − 1.51 × 10^−1^, was wider than in running events, with a steeply rising trend among masters.

As described, we categorized our results into running, jumping, and throwing events, all with three age groups of athletes: (1) youth athletes, including children (i.e., 5–15 years old) and juniors (i.e., 16–19 years old), (2) senior athletes (i.e., 20–35 years old), and (3) masters athletes (i.e., 35–100 years old). Overall, the results show the development of the performance sex gap between the sexes across the lifespan (see Figures 1–3). They also reveal an initial minimum among children, followed by an increase in the gap from the beginning of the junior era that plateaus among senior athletes before rising steeply among masters. For running events, the gap was rather stable in most events but widened across the cohorts in the masters group. In jumping events, the increase was steeper but not as steep as for throwing events, especially for masters, the group with the maximum gap.

### Running events

In running events, the trend was relatively uniform. In early childhood, the performance gap was slight, at approximately 5%, before rising to 8% at the age of 13 years and gradually widening while approaching the senior level. The trendline for senior athletes 20–35 years old, displaying an approximate 10% difference across all events (see Figure 1), was nearly flat. The performance gap then began rising slightly among masters athletes. Among 50–54-year-olds, the performance gap was approximately 12% before gradually increasing in every older age-based cohort until reaching approximately 20% among 75–79-year-olds. Beginning at the age of 80 years, the increase steepened to reach approximately 30% in the oldest age-based cohorts.

Between events, the performance sex gap increased at approximately 55 years of age in the sprinting events (i.e., 100 m, 200 m, and 400 m) and in the 800 m distance run but occurred at a later age in long-distance running events (i.e., 3,000 m, 5,000 m, 10,000 m, and the marathon). The development can be depicted by the following polynomial regression line: 1.09 × 10^−6^×^3^ − 1.33 × 10^−4^×^2^ + 5.93 × 10^−3^× + 2.16 × 10^−2^.

### Jumping events

Although the trend in the performance sex gap in jumping events was comparable to that in running events, the gap was mostly larger overall (see Figure 2). A slight gap beginning in early childhood increased to approximately 9%, albeit with variations, at the approximate age of 10 years. Next, at approximately 12–13 years of age, the gap widened to 12% and continued to widen. Among the youngest senior athletes, the gap widened further to 15% but increased only slightly, to approximately 18%, across all older senior age-based cohorts. Among masters athletes, the gap remained at approximately 18% before rising slightly to approximately 25% among 80–84-year-olds, albeit with some variation, and further rising to approximately 33% among 95–100-year-olds (see Figure 2). However, that final difference may have been influenced by the right-tail data of the pole vault event, which no cohorts participated in beyond the 85–89-year-olds.

Among senior athletes, the long jump showed similarities with the high jump in the performance sex gap, both of which were approximately 15%. The gap in the triple jump, at approximately 17%, was somewhat larger than in those events. However, the widest gap in any jumping event, at approximately 20%, occurred with the pole vault. In that event, the increased gap among masters athletes began at 35 years of age save an outlier among 70–74-year-olds. Even so, the gap remained stable in all other jumping events until the age of 65 years, when it began rising slightly until 80 years of age, after which a steep increase occurred. The development of the performance gap in jumping can be depicted by the following polynomial regression line: 1.69 × 10^−6^×^3^ − 2.51 × 10^−4^×^2^ + 1.21 × 10^−2^× − 1.05 × 10^−3^.

### Throwing events

**Figure 4:**
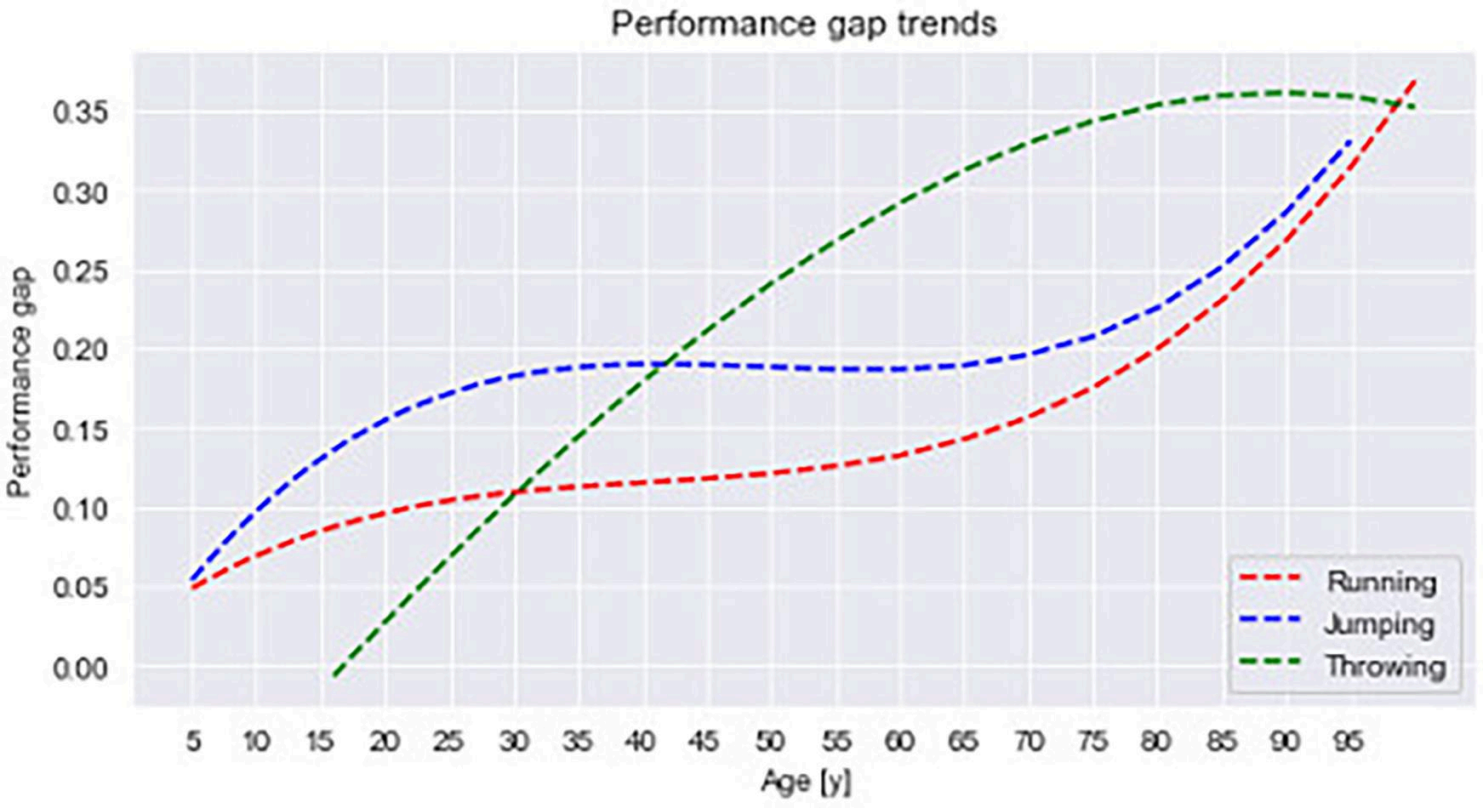
Trendlines in all categories of events: running (red), jumping (blue), and throwing (green). Polynomial regression lines follow the equations: 1.09 × 10^−6^x^3^ − 1.33 × 10^−4^x^2^ + 5.93 × 10^−3^x + 2.16 × 10^−2^ (i.e., for running events), 1.69 × 10^−6^x^3^ − 2.51 × 10^−4^x^2^ + 1.21 × 10^−2^x − 1.05 × 10^−3^ (i.e., for jumping events), and −2.55 × 10^−7^x^3^ − 1.79 × 10^−5^x^2^ + 9.37 × 10^−3^x − 1.51 × 10^−1^ (i.e., for throwing events).

The performance sex gap in throwing events (see Figure 3) differed from that in the other two categories of events in multiple ways. First, the data shown in Figure 3 were not directly comparable with data for the other two categories because they display only children's performance when using senior-level throwing implements beginning at the age of 12 years. The performance of younger athletes using differently scaled implements could not be used because there was no consensus in the data on the scaling of throwing implements used by children (see Figure 4). Second, because the throwing implements represented in the data were scaled, the real performance sex gap may be concealed. Bearing those limitations in mind, it can be argued that, there was a small performance sex gap between boys and girls that rose as they reached the senior level. The event with the largest gap was javelin throw, in contrast to discus throw, where the gap was even negative.

Among senior athletes, the gap was relatively slight in both the shot put and hammer throw and even slightly negative (i.e., women outperformed men) in the discus event. In the javelin, there was a stable gap of approximately 25% across the senior cohorts.

Among masters, the gap increased but at different rates across the different events. On average, the trendline rose steadily before leveling off among 75–79-year-olds. The gap increased from the 15% average at the age of 35 years to approximately 25% at 50 years, 30% at 65 years, and 35% at 80.

Between events, the greatest performance sex gap and the greatest increase in the gap occurred in the javelin throw, which developed similarly to the gaps in the other throwing events from the mentioned 25% among senior athletes to approximately 50% among the oldest masters. The trendline in javelin also differed from the rest of the throwing events by increasing steeply at the beginning of the masters cohorts between 35 and 45 years of age. Another steep increase occurred in hammer throw just after the senior level, from approximately 10% at the age of 35 to 30% among 70–74-year-olds. The development of the gap in shot put, meanwhile, was comparable with that in the hammer throw, with a roughly 10% difference observed among youth and a decrease to approximately 6% among seniors. However, the transfer to the masters cohorts was smoother in the shotput than in the hammer throw, and performance gap remained below 20% until the age of 55 years, when it began increasing. In the discus, the performance gap in juniors and seniors inverted and hovered around 0%, thereby indicating that women's outperformed men at those ages. The gap in discus rose dramatically, from approximately 4% at 45 years of age to 30% at approximately 50 and continued to increase among masters, where the trend became comparable with that of the javelin throw.

All throwing events compared with other events demonstrated a trend, with the curve of the performance sex gap continuing to widen progressively among the masters cohorts. The development of the gap in throwing events can be depicted by the following polynomial regression line: −2.55 × 10^−7^x^3^ − 1.79 × 10^−5^x^2^ + 9.37 × 10^−3^x − 1.51 × 10^−1^.

The trendlines in each category of events, shown in Figure 4, developed similarly in running and jumping events, albeit at different levels, with the magnitude of the performance sex gap being greater in jumping events. Both trendlines also revealed similar stability among seniors and rose throughout the masters cohorts. By contrast, the trendline for throwing differed considerably from the trendlines in the other two categories (see Figure 4).

## Discussion

In our study, we examined the development of the performance sex gap in 18 track-and-field events—an unprecedented number of events in such studies—grouped in three categories (i.e., running, jumping, and throwing) based on similarities in physical demands. Due to important differences in demands between those categories, it was possible to draw more general conclusions based on variations in the performance sex gap across the categories. That said, differences emerged between events within each category but were not big enough to alter our conclusions. Whereas most previous studies have focused on athletes in their active (i.e., senior) careers and have not usually examined children or older athletes (4, 10, 19, 20), we examined age-related differences in the performance sex gap across age cohorts across the entire lifespan. Our results show that the gap changes with age and differs between youth athletes, senior athletes, and masters athletes.

Among young children, the performance sex gap was evident, with a magnitude of approximately 5%, before increasing during adolescence. When boys enter puberty, their levels of testosterone increase dramatically, and secondary changes in their bodies occur. By contrast, girls' development is more modest, and, as a result, the performance gap gradually widened during the late teenage years. Subsequently, among senior athletes, the performance gap was rather stable from approximately 20 to 30 or even 35 years of age, albeit with notable variations across events and event categories. The gap's magnitude was approximately 10% in all running events and even greater in jumping events (i.e., approx. 17%), though with some variation, but smaller in throwing events. That last trend resulted from the downscaling of the weight of thrown implements, in the discus event, for instance, the gap disappeared altogether and even became negative (i.e., women outperformed men). The magnitude of the performance gap in throwing corresponded well with the degree of downscaling, which was greatest in the discus event (i.e., scaled down to 50%), somewhat less in the shot put and hammer throw events (i.e., 55%), and least in the javelin event (i.e., 75%), as detailed in Supplementary Appendix S2. From our results, we can conclude that such scaling affects the performance gap's development across the lifespan.

While the gap was relatively stable during athletes' senior careers, it grew as they aged, although at different speeds depending on the event category. The gap was widest among the oldest athletes, whose maintenance of physiological abilities varies according to sex. By comparison, the difference across event categories was similar among seniors—that is, remained small in the running events, increased in the jumping events, and was truly different in the throwing events because of variously scaled throwing implements. Although the gap among masters was the greatest, evidence suggests that the performance of masters athletes is improving and that the gap is narrowing (4, 27, 61). It is therefore likely that the trend indicated by senior athletes' records will soon occur among masters athletes, which will also cause the performance gap to decrease.

Among adult athletes, the category of throwing events has not been included in any previous studies on sex differences in athletic behavior due to difficulties with making comparisons because the weight of implements is scaled down for women. In our study, such scaling also masked the actual difference between the sexes. Because the scaling currently used in athletics lacks consistency and transparent argumentation, it causes great differences between sporting events. As a result, some events are scaled better than others as seen from the results (e.g., discuss vs. javelin throw, see Table 2). The scaling in the masters category seems far more arbitrary and, in fact, increased the gap relative to other event categories, as shown in Figure 4. The trendlines for running and jumping were similar, except that the difference in jumping was great, likely due to men's superior strength, which enables them to overcome gravity better than women can. The trendline for throwing events affected by the variable scaling between sexes, revealed a completely different trend. Had the scaling been more balanced, the trendline would most certainly be more like the other two, probably with an even wider gap than in running. The effect of improper scaling masks the real performance of women who compete with relatively heavier implements than men; thus, their real performance remains underestimated, which causes their performance to decrease faster than men's with advancing age and thus widens the performance gap. Such underestimation of women's performances may harm the reputation of women's sports. In another concern related to the variable scaling, the lack of a unified specification of throwing implements for younger athletes complicates any comparison of boys' and girls' performance, and thus precludes any acknowledgement of recommendations for training specifically tailored to younger girls.

**Table 2:** Weight of throwing implements used by youth athletes (i.e., children and juniors). Weight is displayed in kilograms except for the javelin (*), for which the units are grams. The weight of the throwing implements is shown as % MAX, meaning the percentage of the seniors’ equipment (i.e., weight of the children's implement/senior weight). A detailed overview of equipment used by youth in various countries appears in Table 3 below.

Event	Sex	Weight	% MAX	Weight	% MAX	Weight	% MAX	Weight	% MAX	Weight	% MAX	Weight	% MAX	Weight	% MAX
Shot put	M	2	28	3	41	4	55	5	69	6	83	7.26	100		
	W	2	50	3	75	4	100								
Hammer	M	2	28	2.5	3.44	3	41	4	55	5	69	6	83	7.26	100
	W	2	50	2.5	63	3	75	4	100						
Javelin* (g)	M	400	50	500	63	600	75	700	88	800	100				
	W	400	75	500	84	600	100								
Discus	M	0.5	25	0.6	30	0.75	38	1	50	1.5	75	1.75	88	2	100
	W	0.5	50	0.75	75	1	100								

Bold values indicate MAX (senior) weight.

**Table 3:** Scaling of throwing implements in children and juniors.

Country	Category	Shot put (kg)		Hammer (kg)		Javelin (g)		Discus (kg)	
		M	W	M	W	M	W	M	W
Australia	U13	3	3	–	–	600	400	0.75	0.75
	U14	3	3	3	3	600	400	1	1
	U15	4	3	4	3	700	500	1	1
	U16	4	3	4	3	700	500	1	1
	U17	5	3	5	3	700	500	1.5	1
	U18	5	3	5	3	700	500	1.5	1
	U20	6	4	6	4	800	600	1.75	1
Canada	U16	4	3	4	3	600	500	1	1
	U18	5	3	5	3	700	500	1.5	1
	U20	6	4	6	4	800	600	1.75	1
Czechia	U13	3	2	–	–	500	400	–	–
	U16	4	3	4	3	600	500	1	0.75
	U18	5	3	5	3	700	500	1.5	1
	U20	6	4	6	4	800	600	1.75	1
France	12–13	3	2	3	2	500	400	1	0.6
	14–15	4	3	4	3	600	500	1.25	0.8
	16–17	5	3	5	3	700	500	1.5	1
	18–19	6	4	6	4	800	600	2	1
Germany	U14	3	3	3	2	400	400	0.75	0.75
	U16	4	3	4	3	600	500	1	1
	U18	5	3	5	3	700	500	1.5	1
	U20	6	4	6	4	800	600	2	1
Netherlands	U9	–	–	–	–	–	–	–	–
	U10	2	2	–	–	–	–	–	–
	U12	2	2	–	–	–	–	–	–
	U14	3	2	–	–	400	400	1	0.75
	U16	4	3	4	3	600	500	1	1
	U18	5	3	5	3	700	500	1.5	1
	U20	6	4	6	4	800	600	1.75	1
Norway	6	2	2	–	–	–	–	–	–
	7	2	2						
	8	2	2						
	9	2	2	2	2	400	400	0.6	0.6
	10	2	2	2	2	400	400	0.6	0.6
	11	2	2	2	2	400	400	0.6	0.6
	12	3	2	2	2	400	400	0.75	0.6
	13	3	2	3	2	400	400	0.75	0.6
	14	4	2	4	3	600	400	1	0.75
	15	4	3	4	3	600	500	1	0.75
	16	5	3	5	3	700	500	1.5	1
	17	5	3	5	3	700	500	1.5	1
	18–19	6	4	6	4	800	600	1.75	1
	20–22	7.26	4	7.26	4	800	600	2	1
UK	U13	3	2.72	–	–	400	400	–	–
	U15	4	3	4	3	600	500	1.25	1
	U17	5	3	5	3	700	500	1.5	1
	U20	6	4	6	4	800	600	1.75	1
USA	D8	2	2	–	–	300	300	–	–
	D10	2.72	2.72			300	300		
	D12	2.72	2.72			450	450	1	1
	D14	4	2.72			600	600	1	1
	D16	5.42	4	6	4	800	600	1.6	1
	D18	5.42	4	6	4	800	600	1.6	1
	U20	6	4	6	4	800	600	1.75	1
WA	U18	5	3	5	3	700	500	1.5	1
	U20	6	4	6	4	800	600	1.75	1
	Senior	7.26	4	7.26	4	800	600	2	1

Australian Athletics (athletics.com.au); Athletics Canada (athletics.ca); Czech Athletic Federation (atletika.cz); French Athletics Federation (athle.fr), German Athletics Association (leichtathletik.de), Royal Dutch Athletics Federation (atletiekunie.nl); Norwegian Athletics Association (Friidret.no); Youth Development League (ukydl.org.uk); Track and Field Athletics (usatf.org) and World Athletics (WA, worldathletics.org). WA) defines categories U18 as any athlete of 16–17 years old an U20 as 18–19 years old on December 31 in the year of the competition (62). Although most countries have adapted the international format of category, some countries distinguish specific categories. For example, the Czech Republic distinguishes mladší žáci (“younger students”), starší žáci (“older students”), and dorostenci (“adults”); France distinguishes “Benjamins”, “Minimes”, “Cadets”, and “Juniors”; and the United States distinguishes “age divisions”. By further contrast, Norway distinguishes two junior age groups: 18–19-year-olds and 20–22-year-olds. In the hammer throw, the length of the wire changes according to the weight of the hammer implement (see Supplementary Appendix S2); however, in Norway, the hammer is defined together with the length of the hammer rope as 2 kg 110 cm, 3 kg 110 cm, 4 kg 119.5 cm, 5 kg 120 cm, 6 kg 121.5 cm, and 7.26 kg 121.5 cm.

### Running events

In the running events, the performance sex gap seemed to gradually evolve, starting at approximately 5% among children and remaining constant at approximately 10% among seniors (see Figures 1, 4), which corresponds well with findings from previous studies (10, 18, 19, 22, 30). After 35 years of age, the gap started to increase but remained at approximately 14% until athletes were 60–65 years old. Thereafter, the gap increased rather rapidly until reaching 26% at the age of 90 years. However, a steep increase in the right tail of the trendline should be considered carefully, for the trend could have been affected by the lower participation rates of older women athletes (19). Although women athletes 70 years old and older today do compete in marathons and half-marathons (60, 63), involvement in masters sports has increased in general (27, 28).

Our results, shown in Figure 1, revealed a slightly smaller performance sex gap in sprinting events than in longer-distance events, which supports previous findings (9). In longer-distance events (i.e., 3,000 m, 5,000 m, and 10,000 m), the gap persisted for longer than in sprinting events and increased less until 65 years of age, and in the marathon, the increase occurred even later, at approximately 70 years of age. While short-distance events rely primarily on pure power generation at high intensity and peak performance occurs in the early 20 s, longer-distance events require endurance that peaks later in life (20) and thus is maintained longer in both sexes. In extreme cases such as marathons and even ultra-distance swimming events, peak performance occurs even later, apparently due to non-physiological factors such as pace, nutritional strategy, and mental resilience (20). The earlier increase in sprinting events may be attributed to postmenopausal changes that occur in women at the approximate age of 50 and affect their maintenance of skeletal muscles, their amount of adipose tissue (1, 39, 64), and, in turn, their power generation. For men, decreases in testosterone levels occur rather gradually at a magnitude of approximately 1% per year beginning in the late 30 s (57), which negatively affects their performance. However, evidence showed that in healthy middle-aged men, the decrease was not significant and even older men showed normal levels of testosterone (i.e., above 400 ng/dl); moreover, that the positive effect on maintenance of testosterone levels is fitness and also that BMI appeared to negatively affect the testosterone levels (65).

Given running's widespread popularity and high participation rate, running could be regarded as somewhat of a reference for the development of the performance sex gap. The trendline of running events rose in the right tail of the curve among masters athletes, thereby demonstrating the effect of aging due to changes in hormonal regulation. Although our study design had a limitation due to comparing only the best athlete in each age-based cohort, when Thibault et al. (10) compared the best 10 results in athletics events in the Olympic Games with the top performance only, the difference was only approximately 1%, similarly to what Knechtle et al. (8) found in their comparison of age cohorts of 50 top performers in running events. Thus, our data are arguably representative of top performance.

### Jumping events

The performance sex gap was greater in jumping events than in running events, probably due to the higher demands on the upper body in jumping events. Whereas running requires primarily the power of the lower body, specifically the legs, jumping involves, apart from the lower body, upper-body power because athletes need to overcome gravity and lift their body mass into the air. Women's lower-body muscle strength is only approximately 60%–70% of men's (33). An even larger discrepancy exists between women's and men's upper-body strength, for women have only 50%–60% of men's (1, 19, 33, 51).

The gap evolved in early childhood and increased to roughly 9% at the age of 10 years which was slightly larger than reported in recent literature (21, 30), possibly due to the variable development in the pole vault that affected the trendline. However, again, due to our study design, the results in younger athletes need to be considered with caution. The gap was more evident at the age of 13 years, when it grew to approximately 12%, and increased in all jumping events, which corresponds well with the onset of puberty in males. Then, during the late teenage years, the gap widened to 15% (see Figure 2) and slightly increased to roughly 18% through the end of the senior period. The increase seems to mostly stem from the bigger gap in the pole vault event, in which peak age occurs sooner among women than men (66) and, due to the power demands, may be more difficult to perform for masters women, a trend that reflects men's and women's different peak ages in power-demanding events (20). In the masters group, the performance gap remained constant until the age of 65–70 years, when the ability to maintain the same performance drops.

All jumping events showed a similar trend except for the pole vault, which seemed to affect the trendline of jumping events in general. Omitting the pole vault made the performance sex gap in jumping events follow a relatively similar trend to running events, only in jumping events, the trendline remained flat for a shorter period for seniors and for masters until the approximate age of 60 years. At that point, it rose and, from 75 years onward, rose again, likely due to declining levels of testosterone and consequent power loss among masters athletes. The pole vault's trendline showed the greatest gap, with high differences between the groups of age-based cohorts. The event requires initial high-speed, explosive running followed by a rapid transmission of the kinetic energy to the vertical force used to plant the pole and lift the body (67). The event is also highly demanding for the upper body, which favors men's physiology. Simultaneously, the tradition of pole vaulting is older among men; women's pole vault was introduced to the Olympics Games only in 2000 (68).

In contrast to our findings, previous studies have highlighted the long jump as the event with the greatest performance sex gap (i.e., 18.8%), primarily because the pole vault could not be compared due to its recency as a women's event (10). Our study showed that the long jump exhibits similarities with the high jump and the triple jump, and that those events are comparable. All three require high-velocity performance and strength in full-body synergies (69) associated with the task constraints; however, the difficulty of the pole vault between the sexes is the greatest, as was the performance gap in our study.

### Throwing events

In throwing events, the general trend differed from both running and jumping events in that there was seemingly no performance sex gap across senior athletes. Even so, that trend was obviously an artifact due to the downscaling of women's thrown implements in weight and size, to between 50% of men's implements in the discus throw and to 55% in the shot put and hammer throw, all among seniors (see Table 1). That much scaling in throwing events seems to be sufficient to even out men's and women's performances, thereby indicating that the scaling is fair.^1^ The gap in our study was similar, as well as rather slight, in the shot put and hammer throw events (Figure 3). In the discus throw, women even outperformed men slightly in absolute length thrown, which is logical considering the scaling. The case differed slightly for the javelin throw. Women's javelins weigh 75% of men's, and the center of gravity is shifted relative to men's. Consequently, the gap was larger in the javelin throw, at approximately 25% among seniors, than in the other throwing events. Center of gravity aside, the result indicates that scaling down thrown implements to only 75% is not enough to cancel out men's strength-based advantage. Nevertheless, the stated facts become useful information precisely due to such variable scaling. Pedersen and Stalsberg^1^ have argued that the magnitude of scaling of implements in throwing events seems fair relative to the biological differences between men and women, for they result in absolute performances that are highly similar between the sexes. Thus, incorporating that knowledge, we can estimate the real sex-based difference according to the magnitude of the scaling—a difference in percent that would equal the percent scaling. Furthermore, we can consider absolute differences in performance throughout the lifespan in light of such scaling and, from there, estimate the relative differences in performance. In so doing, we can evaluate the fairness of the scaling in older age groups (i.e., masters) and for younger athletes, as well as assess the gap in throwing events by taking the scaling into account. In that light, it seems reasonable that the gap did not change across senior athletes. It is also logical that the gap has been shown to subsequently increase in past research (70).

During adolescence, from our results of youth older than 12 years old, the performance sex gap began widening as boys entered puberty and progressively increased until stabilizing at approximately 20 years of age. That progression makes sense because, as mentioned, the difference between sexes increases year by year during puberty due to the rapid increase in testosterone in boys (49). However, due to the mentioned absence of unified global scaling rules, we did not compare the performance of children less than 12 years old in the throwing events. In a recent study, Brown et al. compared sex-based differences in field events (i.e., javelin throw and shot put) in younger children competing with the same weight implement. Their results reported rather large differences (71); however, the participating athletes varied in their performance, and the specification of the subjects' ages is unclear. As with adults, however, the difference was greatest in the javelin throw. In our study, the smallest difference was in the discus throw, most likely for the same reasons as among adults: that the scaling of thrown implements varies, which has to be considered when making comparisons between the sexes. However, as among children, scaling compromises detailed comparison and makes it difficult to pinpoint relative differences between the sexes. Major international sports authorities (e.g., European Athletics and World Athletics) have not specified common guidelines for throwing implements in younger children athletes (i.e., 5–15-years-olds); thus, each country applies its own rules for each age group (i.e., U12 in the Netherlands but U13 in Czechia; see Table 3) which makes further comparison difficult and inconsistent. Moreover, age groupings in youth categories vary worldwide. In Europe, the categories U18, U20, and U23 are common; however, in the United Kingdom, United Kingdom Athletics (UKA.org.uk) categorizes youth athletes as U13, U15, U17, or U20, and in the United States, the description varies from state to state (Table 3). Therefore, to enable comparison, further consistent specification of the rules of the throwing implements and categorization for younger athletes on the international level is required.

The performance sex gap in throwing events increased rapidly among masters athletes after the age of 35 to 50 years of age, after which the increase was far less steep. An exception was the discus throw, in which the gap did not increase until the age of 45 years. In fact, the gap increased only slightly between ages of 50 and 80 years in the shot put and hammer throw, and the trend was similar in the discus and javelin throw, only the gap was wider (see Figure 3). After the age of 80 years, the results should be interpreted with some caution because there were fewer records for those ages. However, as with the junior age groups, much can be explained by the different scaling between the sexes. As evident from Table 1, the relative weight of implements changes several times during the masters years, and the change is not particularly regular. Women throw implements weighing everywhere from 50% of men's (i.e., slightly less than the 55% among seniors) in the shot put and hammer events at 50–59 years of age and 70–79 years of age, to 100% of men's in the javelin at 70–74 years and after 80 years and, in the discus event, at 60–74 years of age. Thus, with few exceptions, women throw heavier implements than men throughout their masters careers compared with their senior careers. The greatest disparity in the shot put and hammer throw occurs among 70–74-year-olds; men put with 55% of their maximal shot weight, whereas women put with 75% of theirs. In the javelin throw, at 70–74 years of age, men throw 63% of their maximum javelin weight, whereas women throw 83% of theirs. Last, in the discus throw, the greatest disparity occurs among 60–64-year-olds (i.e., 50% max. in men and 100% max. in women). Throughout the masters career, as shown in Table 2, men's implements are gradually scaled down in weight as age increases, while women's implements are scaled down much less and often kept at the same absolute weight for much longer. On the topic, the most striking finding was that women throw a discus of the same weight as in their senior careers until the age of 75 years, whereas men's discus is scaled down to 75% at 50 years of age and further reduced to 50% at 60 years. The weight of the discus was found to be fairly scaled for seniors because women can throw a 1 kg implement equally far as men throw a 2 kg one,^1^ and that weight difference between the sexes corresponds well with their different upper-body strengths.

Such unfair scaling makes women's performance look poorer than men's, which has several consequences. First, it affects women's sports; women are already considered to be weaker, and women's sports are often ridiculed and cast as being less consequential than men's (72). Second, women's sports are already less reported on in written media, broadcasting, and news media than men's sports (73). Third, the real performance-related development in throwing is concealed, and thus the performance gap is overestimated. In response, setting fair conditions by scaling down women's throwing implements would enable spectators to observe real performances by both sexes and their development over time and, for those reasons, should be prioritized.

### Summary

Our study showed that the development of the performance sex gap in three categories of athletic events varies in size and changes over time with age. The most stable gap, at 10%, emerged in running events. The gap in jumping events was larger, at approximately 17%, with the pole vault being notably different. That event could be regarded as the most challenging event for women and, in any case, disproportionately influenced the trendline.

The third category, throwing, varied the most and was affected by the current scaling of throwing implements. Although the definition of *scaling* is based on formal documents of athletic authorities (62), the literature shows no detailed argumentation about or even description of the way in which scaling is developed. As our study shows in some events the scaling is tailored more adequately than in other events, also within the changing periods of life. The performance sex gap in throwing events was slight among seniors, specifically in well-scaled events such as the discus throw; however, among masters, the gap increased sharply. Our results indicate that such scaling in the masters years is unfair to women, and we also argue that it underestimates women's performance. Had the scaling not affected performance, it would primarily change as a function of age, and the trendlines for the different event categories would converge to a higher degree. Furthermore, there is a need for a clear and consistent definition of the scaling of throwing implements of younger children athletes (e.g., 5–15 years old).

In all, our study has illuminated variations in the development of the performance sex gap across the lifespan in and between three categories of athletic events. Moreover, it revealed another variable that dramatically affects the performance gap in throwing events: the scaling of thrown implements.

## Data Availability

The original contributions presented in the study are included in the article/Supplementary Material, further inquiries can be directed to the corresponding author.

## Footnotes

^1^Pedersen AV, Stalsberg R. Fair comparison of men's and women's athletic performances: a natural experiment including performance ratios between the sexes across non-scaled and scaled sports events. *Res Sq.* (2025) [In preparation].
