# Supplementary material for: The development of the performance sex gap in track-and-field events across the lifespan
Source: Front Sports Act Living. 2025 Oct 23;7:1659762. doi: 10.3389/fspor.2025.1659762 (PMC12589021; doi:10.3389/fspor.2025.1659762)
Supplement: Supplementary file 1 [file Supplementaryfile1.docx]

**Supplementary files**

**S1 Appendix 1 Data resources**

- World records in athletics, compiled by World athletics, World records held outdoors described over the years in the elite senior category, U20, U18 – available from <https://www.worldathletics.org/records/by-category/world-records>
- Master athletes’ records, compiled by World Master Athletics, tracking of the world records in master athletes (35-110 years old), 5-year cohorts – available from <http://www.worldmastersathletics.org//aboutus.htm> , the version used was updated 4. 10. 2022
- Children and youth international records, compiled by Dominique Eisold, summarizes data from 61 countries in 5-19 years old athletes – available from <http://age-records.125mb.com>, lastly updated 10. 9. 2022
- Masters athletes records, compiled by Wellington Athletics, displays records for Wellington, New Zealand and World records, available from – <http://www.wellingtonmastersathletics.org.nz/rec/rec_marathon.html>
- Records in European athletics, complied by European Athletics, available from <https://www.european-athletics.com/>
- Finnish records database Tilastopaja, a compilation of world records in senior and youth category- available from <https://www.tilastopaja.info/>

**S2 Appendix 2 Technical specification of throwing implements**

| **Shot put** | Weight (kg) | 3 | 4 | 5 | 6 | 7.26 |
| --- | --- | --- | --- | --- | --- | --- |
|  | Diameter of the head (mm) | 85-110 | 95-110 | 100-120 | 105-125 | 110-130 |
| **Hammer throw** | Weight (kg) | 3 | 4 | 5 | 6 | 7.26 |
|  | Diameter of head (mm) | 85-100 | 95-110 | 100-120 | 105-125 | 110-130 |
|  | Length (mm) | 1195 | 1195 | 1200 | 1215 | 1215 |
| **Javelin throw** | Weight (g) | 500 | 600 | 700 | 800 |  |
|  | Length (mm) | 2000-2100 | 2200-2300 | 2300-2400 | 2600-2700 |  |
|  | Diameter of shaft (mm) | 20-24 | 20-25 | 23-28 | 25-30 |  |
| **Discus throw** | Weight (kg) | 1 | 1.5 | 1.75 | 2 |  |
|  | Diameter outside of metal rim (mm) | 180-182 | 200-202 | 210-212 | 219-221 |  |
|  | Thickness of metal plate, central part (mm) | 37-39 | 38-40 | 41-43 | 44-46 |  |

*Detailed specification of scaled throwing implements defined by WA, reference: World Athletics IAAF, Technical rules, C2.1, 2020 (62).*
